# Beyond the Low Frequency Fluctuations: Morning and Evening Differences in Human Brain

**DOI:** 10.3389/fnhum.2019.00288

**Published:** 2019-08-27

**Authors:** Magdalena Fafrowicz, Bartosz Bohaterewicz, Anna Ceglarek, Monika Cichocka, Koryna Lewandowska, Barbara Sikora-Wachowicz, Halszka Oginska, Anna Beres, Justyna Olszewska, Tadeusz Marek

**Affiliations:** ^1^Neuroimaging Group, Malopolska Centre of Biotechnology, Jagiellonian University, Krakow, Poland; ^2^Department of Cognitive Neuroscience and Neuroergonomics, Institute of Applied Psychology, Jagiellonian University, Krakow, Poland; ^3^Chair of Radiology, Medical College, Jagiellonian University, Krakow, Poland; ^4^Department of Psychology, University of Wisconsin—Oshkosh, Oshkosh, WI, United States

**Keywords:** resting state-fMRI, diurnal (circadian) rhythm, morning and evening chronotypes, ALFF/fALFF, functional connectivity

## Abstract

Human performance, alertness, and most biological functions express rhythmic fluctuations across a 24-h-period. This phenomenon is believed to originate from differences in both circadian and homeostatic sleep-wake regulatory processes. Interactions between these processes result in time-of-day modulations of behavioral performance as well as brain activity patterns. Although the basic mechanism of the 24-h clock is conserved across evolution, there are interindividual differences in the timing of sleep-wake cycles, subjective alertness and functioning throughout the day. The study of circadian typology differences has increased during the last few years, especially research on extreme chronotypes, which provide a unique way to investigate the effects of sleep-wake regulation on cerebral mechanisms. Using functional magnetic resonance imaging (fMRI), we assessed the influence of chronotype and time-of-day on resting-state functional connectivity. Twenty-nine extreme morning- and 34 evening-type participants underwent two fMRI sessions: about 1 h after wake-up time (morning) and about 10 h after wake-up time (evening), scheduled according to their declared habitual sleep-wake pattern on a regular working day. Analysis of obtained neuroimaging data disclosed only an effect of time of day on resting-state functional connectivity; there were different patterns of functional connectivity between morning (MS) and evening (ES) sessions. The results of our study showed no differences between extreme morning-type and evening-type individuals. We demonstrate that circadian and homeostatic influences on the resting-state functional connectivity have a universal character, unaffected by circadian typology.

## Introduction

Human’s performance and most biological functions express rhythmic fluctuations across a 24-h-period: an increase during the day and a decrease at night (Carrier and Monk, 2000; Persson and Persson, 2018). This phenomenon is believed to originate from homeostatic and circadian sleep-wake regulatory processes (Borbély, 1982; Roenneberg and Merrow, 2005; Schmidt et al., 2012). As it allows organisms to adjust to cyclic availabilities, it affects cognitive functions and a wide range of neurobehavioral events (Schmidt et al., 2007). Circadian variations were found in several cognitive tasks, including attention, memory, verbal, arithmetic, motor and reaction time (Kuriyama et al., 2003; Ramírez et al., 2006; Edwards et al., 2007; Tassi et al., 2012). Results of various neuropsychological tests suggest that the activity of specific brain areas also may be modulated according to the time of day (Schmidt et al., 2007; Valdez et al., 2012).

Chronotypes are relatively stable traits of the subjective diurnal rhythm of activity. Traditionally, they refer to the subjective morning vs. -evening preferences, i.e., the self-reported “feeling best” and “best performance” times of day. In evening-type people, as compared to morning-type ones, phases of physiological circadian rhythms are shifted toward later hours (e.g., Kerkhof and Van Dongen, 1996; Gibertini et al., 1999; Bailey and Heitkemper, 2001). The time course of circadian rhythms in cognitive performance differs significantly based on chronotype (Horne et al., 1980; Schmidt et al., 2007; Valdez et al., 2012). Only a handful of functional magnetic resonance imaging (fMRI) studies regarding circadian rhythms were performed so far. For example, Gorfine and Zisapel (2009) showed differences in brain activation patterns during night vs. afternoon hours, while Marek et al. (2010) found time-of-day variations in neural activity of the orienting attentional system. Diurnal fluctuations in performance-related brain activity were also observed in studies employing chronotype-based paradigms (e.g., Gorfine et al., 2007; Fafrowicz et al., 2009; Schmidt et al., 2009, 2012, 2015; Vandewalle et al., 2009, 2011; Peres et al., 2011).

All above-mentioned studies concerned particular neuropsychological functions examined by performing specific tasks. Resting-state networks are mostly believed to remain stable throughout the day (Biswal et al., 2010; Byrne and Murray, 2017). An opposite view was postulated by Blautzik et al. (2013) and Park et al. (2012), who argued that the level of stability varies over a period of 1 day across different resting state networks, ranging from highly rhythmic to stable. Jiang et al. (2016) found that widespread brain areas exhibit diurnal variations in resting-state. However, the study’s sample size was relatively small (16 participants), and neither the subjects’ sleep-wake pattern nor chronotypes were controlled, which could have affect the results. In our research we used the chronotype-based paradigm (Schmidt et al., 2007). Thus, the goal of this study was to investigate circadian fluctuations of functional networks using resting-state fMRI (rs-fMRI).

Twenty-nine subjects with an extreme morning chronotype (M-type) and 34 subjects with an extreme evening chronotype (E-type) were evaluated during morning (MS) and evening (ES) sessions, after which we conducted the seed-to-voxel analysis using ALFF-based FC analysis, choosing the brain regions as seeds based on the study of Jiang et al. (2016).

## Materials and Methods

### Participants

Online advertisement on the Jagiellonian University website and Facebook that were targeted at young, healthy individuals were the primary recruitment method for the study. Five-thousand three-hundred and fifty-four volunteers participated in the first stage of selection. All volunteers were asked to complete a sleep-wake assessment (individual sleep need, ideal vs. real bedtimes and waketimes), diurnal preference as measured by the Chronotype Questionnaire (Oginska et al., 2017), night sleep quality as measured by the Pittsburgh Sleep Quality Index (PSQI; Buysse et al., 1989), and daytime sleepiness as measured by the Epworth Sleepiness Scale (ESS; Johns, 1991). Individuals reporting sleep problems or excessive daytime sleepiness were excluded from the study, as determined by the cut-off points from the PSQI (≤5 points) and ESS (≤10 points) questionnaires. Out of the 451 participants who were identified as exhibiting extreme morning or evening chronotypes, 63 healthy, young participants (38 women) were selected for the study. The low selection percentage (14%) was caused by the fact that there are very few extreme morning type people in the general population of young, healthy individuals. All participants needed to have a regular time-of-day schedule without sleep debt (length of sleep between 6 and 9 h per night). Specific selection criteria included: age between 19 and 35 years, right-handedness, no neurological or psychiatric disorders, no significant vision defects, and no MRI contraindications.

All subjects were right-handed as indicated by the Edinburgh Handedness Inventory (Oldfield, 1971). During the selection process, candidates were interviewed about drug, alcohol or nicotine dependence by a clinical psychologist. Subjects, who were dependent on any substance specified above were excluded from the study. Further exclusion criteria included shift work and having been on a flight passing more than two time zones within the past 2 months. Participants were requested to maintain a regular sleep-wake schedule 1 week prior to fMRI scanning, which was controlled using MotionWatch 8 actigraphs. The regular sleep-wake schedule matched the individuals sleep need. Participants wore actigraphs the week preceding the study as well as the days of brain imaging to control sleep length and quality. Furthermore, the night before the morning session, subjects slept in rooms located in the same building, as the MR laboratory. MRI acquisition was postponed in cases where sleep pattern was disrupted. The actigraphy data allow us to find no differences between M- and E-types in any of the parameters derived from actigraphy except for one, which was hour differences in bedtimes and waking times. Demographics, questionnaires and actigraphy results are provided in the Supplementary Material Table S1. Subjects abstained from alcohol (48 h) and caffeine (24 h) before each fMRI session. During experimental days participants could engage in non-strenuous activities. The study was conducted from November 2016 until January 2018 in MR laboratory, Malopolska Centre of Biotechnology, Jagiellonian University, Krakow, Poland and was approved by the Institute of Applied Psychology Ethics Committee of the Jagiellonian University. Informed, written consent was provided by all participants in accordance with the Declaration of Helsinki.

Resting-state fMRI scans were acquired for MS between 8:00 AM and 9:00 AM (M = 8:21 AM; SD = 10 min) for M-type participants, and between 9:20 AM and 10:20 AM (M = 9:41 AM; SD = 14 min) for E-type participants; for ES between 5:00 PM and 6:00 PM (M = 5:19 PM; SD = 14 min) for M-type participants, and between 6:20 PM and 7:20 PM (M = 6:43 PM; SD = 20 min) for E-type participants. Session order was counterbalanced across participants.

## Methods

### MRI Data Acquisition

MRI data was acquired using a 3T Siemens Skyra MR System. A sagittal 3D T1-weighted MPRAGE sequence was used to obtain anatomical images. 10-min functional resting-state blood-oxygenation-level-dependent (BOLD) images were acquired using a gradient-echo single-short echo planar imaging sequence with the following parameters: repetition time (TR) = 1,800 ms; echo time (TE) = 27 ms; field of view (FOV) = 256 mm; slice thickness = 4 mm; voxel size = 4 mm^3^, with no gap, using a 64-channel coil. A total of 34 interleaved transverse slices and 335 volumes were acquired. During the resting state procedure, subjects were instructed to keep their eyes open, think of nothing, and not to fall asleep. Subjects’ awakeness was monitored using an eye tracking system (Eyelink 1000, SR research, Mississauga, ON, Canada).

### fMRI Data Pre-processing

The rs-fMRI data was processed using MATLAB version R2016a (The MathWorks Inc., Natick, MA, USA) and a statistical parametric mapping software (SPM12; Wellcome Trust Centre for Neuroimaging, UCL, London, UK). Scans were then slice-timed corrected, realigned by inclusion of field maps, which were created using “FieldMap Toolbox” included in SPM 12. After realignment, motion parameters were estimated for each volume of every subject. No participant needed to be excluded due to extensive movements, defined as exceeding a 3° rotation or 3 mm translation on any axis. Following motion correction, each individual’s structural T1-weighted image was co-registered and spatially normalized to Montreal Neurological Institute (MNI) space, as well as resampled to 3 mm^3^ isotropic voxels using B-Spline Interpolation. Finally, the normalized volumes were smoothed using 6-mm FWHM Gaussian kernel.

### ALFF-Based FC Analysis

Pre-processed data underwent connectivity analyses using the CONN: Functional Connectivity Toolbox (CONN v17.f). Confounding effects of white matter, cerebrospinal fluid, and six motion parameters obtained from realignment pre-processing steps were removed by linear regression. BOLD data was then band-pass filtered (0.008–0.09 Hz) to remove high-frequency noise, linear detrended, and despiked. First level functional connectivity analysis was performed using seed-to-voxel whole brain correlations at an uncorrected level (*p* < 0.001). Bivariate Pearson correlation coefficients between the time series of seeds and the rest of the voxels in the brain were extracted and then transformed into Fisher *Z*-scores. Regions of Interest (ROIs) were chosen based on a previous report by Jiang et al. (2016) using an ALFF-based FC approach (Tadayonnejad et al., 2015). All ROIs were derived from the Automated Anatomical Labeling (AAL)-90 atlas (Tzourio-Mazoyer et al., 2002). Description of the selected ROIs and their MNI coordinates are provided in Supplementary Material Table S2.

### Statistical Analyses

First-level, subject-specific connectivity maps for each seed were used in a second-level analysis. We used three different statistical approaches to verify our research hypotheses:

(1) a two-sided paired *t*-test implemented in CONN was performed, in order to investigate differences in seed-to-voxel connectivity between MS and ES for each ROI and the rest of the brain.
(2) two sample *t*-test implemented in CONN was used to evaluate possible differences between M-type and E-type participants in seed-to-voxel connectivity between each ROI and the rest of the brain.
(3) whole-brain voxel-wise 2 (between-subject factor: morning and evening chronotype) × 2 (within-subject factor: morning and evening session) repeated measures ANOVA was performed to evaluate the time of the day × chronotype effect on FC between selected ROIs and the rest of the voxels in the brain.

Results were then exported to SPM in order to identify significant clusters. Based on previous studies (Ichesco et al., 2012; Fallon et al., 2016; Zhu et al., 2017), voxelwise statistics throughout the whole brain were performed at an uncorrected (*p* < 0.001) level, and clusters which survived a false discovery rate (FDR) correction at a cluster level of *p* < 0.05, were reported.

## Results

### Seed-to-Voxel Analysis in Relation to Time of the Day

**Figure 1:**
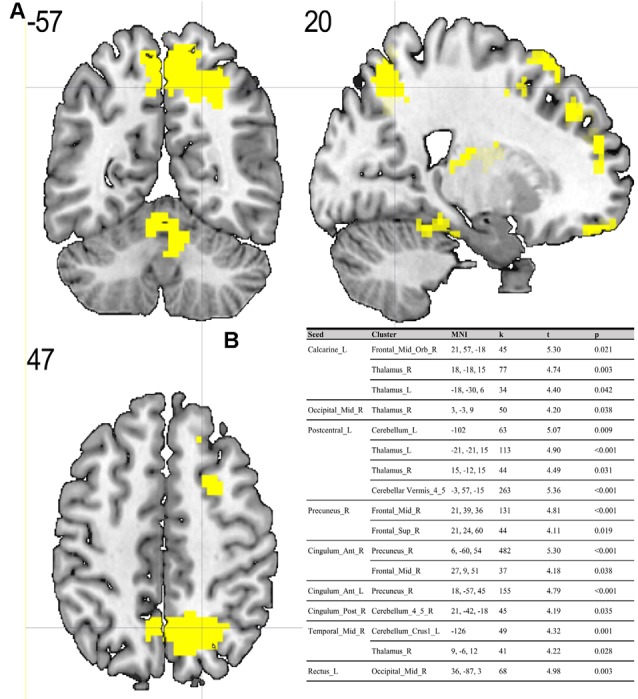
**(A)** Results of two-sample *t*-test on functional connectivity maps with MS > ES contrast. Yellow areas depict increased functional connectivity in the morning session (MS) compared to the evening session [ES; *p* < 0.05; false discovery rate (FDR) corrected on cluster level]. **(B)** Table representing seeds with the presence of at least one positively correlated cluster with significant (*p* < 0.05; FDR corrected on cluster level) differences between morning and evening sessions.

**Figure 2:**
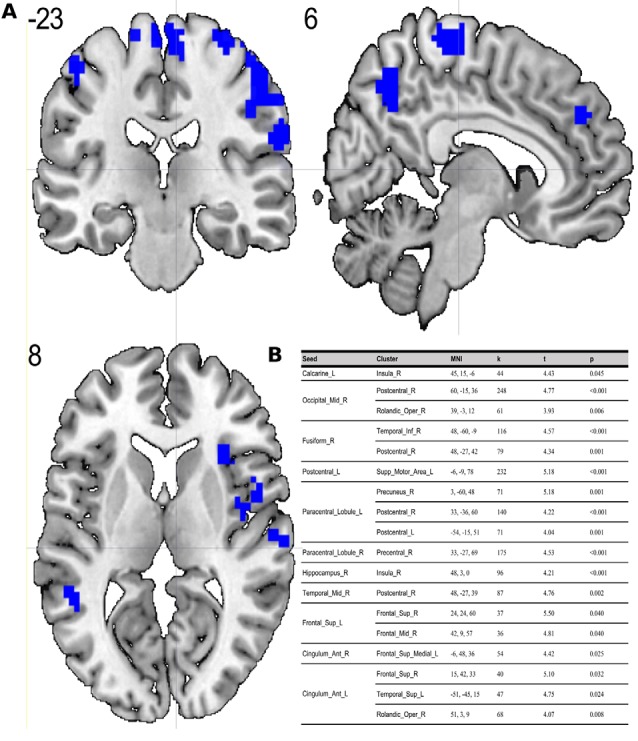
**(A)** Results of two-sample *t*-test on functional connectivity maps with ES > MS contrast. Blue areas depict increased functional connectivity in the evening session compared to the morning session (*p* < 0.05; FDR corrected on cluster level). **(B)** Table representing seeds with the presence of at least one positively correlated cluster with significant (*p* < 0.05; FDR corrected on cluster level) differences between evening and morning sessions.

Compared to the ES, the MS showed significant (*p* < 0.05; FDR corrected on cluster level) differences in multiple brain regions—see Figures 1, 2.

### Seed-to-Voxel Analysis in Relation to Chronotype

We did not find any significant differences (*p* > 0.05) between extreme M-type and E-type in seed-to-voxel connectivity between each ROI and the rest of the brain.

### Seed-to-Voxel Analysis in Relation to Time of the Day × Chronotype

There were no significant chronotype × time of the day interactions at any cluster (*p* > 0.05; FDR corrected on cluster level) at neither morning nor evening session.

## Discussion

To the best of our knowledge, our study is the first depicting functional connectivity in subjects with extreme chronotypes using ALFF-based FC analysis. The resting state functional connectivity patterns were evaluated in a limited number of previous studies which provided inconclusive results. In our article, we have chosen to concentrate on clusters which were identified in a article of Jiang et al. (2016), reported as having both increased and decreased ALFF values. We have focused on ALFF instead of ReHo analysis due to the distinct nature of those two separate analyses. While REHO reflects the local synchronization of spontaneous neural activity between neighboring voxels, ALFF measures the fluctuation amplitude of spontaneous neural activity at the single-voxel level (global), being more suitable for a seed-to-voxel approach (An et al., 2013). Our results showed that diurnal differences in amplitudes of low frequency fluctuations are also involved in seed-to-voxel functional connectivity related to the time of day. Previous study by Hodkinson et al. (2014) that, focused on the functional connectivity and regional cerebral blood flow confirmed that the default mode network (DMN) decreases its integration in the afternoon compared to in the morning. Blautzik et al. (2013) revealed that daily modulation of resting-state connectivity patterns fluctuates from highly rhythmic (DMN and sensorimotor regions) to stable (frontal-cortical areas). Only one recently published article by Horne and Norbury (2018) explored the impact of chronotype on functional connectivity in the brain. Horne et al.’s study results suggest that evening-oriented participants showed reduced connectivity within the DMN network. On the contrary, our results state that the influence of the time of day is stronger than that of the chronotype on functional organization of the brain. No diurnal differences between extreme chronotypes were found in the areas which are a part of DMN. Therefore, our results are not in line with the outcomes of Horne and Norbury (2018). Our seed-to-voxel analysis revealed functional connectivity differences between morning and evening sessions, between regions involved in important resting-state networks. Namely, in the morning compared to the evening session, we found increased connectivity between regions which are part of visual and sensorimotor networks and the thalamus. The thalamus is a critical structure for the integration and processing of sensory and motor information. It is a relay station for visual, auditory and sensorimotor pathways on the way to the cerebral cortex (Huguenard and McCormick, 2007). The thalamus also plays a role in homeostatic sleep pressure, sleep regulation and wakefulness, due to its many nonphotic and photic inputs into the suprachiasmatic nucleus (SCN; Jan et al., 2008). Schmidt et al. (2009) showed that global alertness in the evening was associated with a larger thalamus response in M-type individuals compared to E-type individuals. In addition, our work showed increased connectivity between the left postcentral gyrus, right middle temporal gyrus and anterior cingulate cortex and parts of the cerebellum during the MS as compared to the ES. It is well known, that the cerebellum is involved not only in motor activities, but also in a variety of cognitive functions (Buckner, 2013). The increased functional connectivity we found between the cerebellum and postcentral gyrus may indicate greater involvement of the sensorimotor system early in the morning. A previous study by Tüshaus et al. (2017) showed decreased connectivity of the sensorimotor network as the amount of time awake increases, which is in line with our results.

It may be concluded that the time of day effect is stronger than the chronotype effect on resting-state functional connectivity in the brain. The results of our study showed that circadian and homeostatic influences on resting-state functional connectivity have a universal character and it is unaffected by circadian typology.

## Data Availability

All datasets generated for this study are included in the manuscript and/or the Supplementary Files.

## Ethics Statement

### Human Subject Research

The studies involving human participants were reviewed and approved by Jagiellonian University Ethic’s Commission. The patients/participants provided their written informed consent to participate in this study.

## Author Contributions

MF was the study coordinator and contributed to the study idea, fMRI data acquisition, discussion of analysis and results, as well as writing and revision of the manuscript. BB contributed to the scripting of data preprocessing, data analysis, discussion of analysis and results, as well as writing and revision of the manuscript. AC contributed to the analysis of demographics, questionnaires and actigraphy results, discussion of analysis and results, as well as writing and revision of the manuscript. MC, KL, BS-W, and AB contributed to fMRI data acquisition and revision of the manuscript. HO contributed to the analysis of demographics, questionnaires and actigraphy results, as well as revision of the manuscript. JO contributed to the revision of the manuscript. TM contributed to the study idea, the discussion of analysis and results, and revision of the manuscript.

## Conflict of Interest Statement

The authors declare that the research was conducted in the absence of any commercial or financial relationships that could be construed as a potential conflict of interest.

## Abbreviations

AAL: Automated Anatomical Labeling
BOLD: blood-oxygenation-level-dependent
DMN: default mode network
ES: evening session
ESS: Epworth Sleepiness Scale
E-type: extreme evening chronotype
FDR: false discovery rate
fMRI: functional magnetic resonance imaging
FOV: field of view
MNI: Montreal Neurological Institute
MS: morning session
M-type: extreme morning chronotype
PSQI: Pittsburgh Sleep Quality Index
ROI: region of interest
rs-fMRI: resting-state fMRI
SCN: suprachiasmatic nucleus
TE: echo time
TR: repetition time.
