# Supplementary material for: Beyond the Low Frequency Fluctuations: Morning and Evening Differences in Human Brain
Source: Front Hum Neurosci. 2019 Aug 27;13:288. doi: 10.3389/fnhum.2019.00288 (PMC6718916; doi:10.3389/fnhum.2019.00288)
Supplement: Supplementary file 1 [file Data_Sheet_1.ZIP › Table 1.docx]

Table 1. Demographics, questionnaires and actigraphy results (M-type – extreme morning type, E-type – extreme evening type, ME – morningness/eveningness scale from Chronotype Questionnaire, AM – amplitude scale from Chronotype Questionnaire, ESS – Epworth Sleepiness Scale, PSQI – Pittsburgh Sleep Quality Index, p<0.05)

| **variables (mean ± SD)** | **M-type (N=29)** | **E-type (N=34)** | **sign.** |
| --- | --- | --- | --- |
| sex (M/F) | 17/12 | 13/21 | ns |
| age (years) | 24.86 ± 3.83 | 24.7 ± 3,10 | ns |
| ME | 16.03 ± 2.37 | 28.65 ± 2.10 | * |
| AM | 21.72 ± 3.56 | 22.62 ± 3.00 | ns |
| ESS | 6.62 ± 2.58 | 7.88 ± 4.10 | ns |
| PSQI | 2.79 ± 1.11 | 3.24 ± 1.18 | ns |
| declared waketime (hh:mm) | 6:52 ± 57 min | 7:38 ± 53 min | * |
| declared bedtime (hh:mm) | 23:17 ± 55 min | 00:11 ± 49 min | * |
| declaredlengthofperfectsleep(hh:mm) | 08:32 ± 45 min | 08:38 ± 48 min | ns |
| actigraphy-derivedwaketime(hh:mm) | 7:38 ± 68 min | 8:33 ± 75 min | * |
| actigraphy-derivedbedtime(hh:mm) | 23:57 ± 63 min | 00:57 ± 64 min | * |
| actigraphy-derived length of real sleep (hh:mm) | 7:35 ± 32 min | 7:27 ± 55 min | ns |
