# Supplementary material for: Beyond the Low Frequency Fluctuations: Morning and Evening Differences in Human Brain
Source: Front Hum Neurosci. 2019 Aug 27;13:288. doi: 10.3389/fnhum.2019.00288 (PMC6718916; doi:10.3389/fnhum.2019.00288)
Supplement: Supplementary file 1 [file Data_Sheet_1.ZIP › Table 2.docx]

Table 2. Description of the selected ROIs and their MNI coordinates (AAL- Automated Anatomical Labeling, MNI – Montreal Neurological Institute)

| **Region (AAL)** | | **Center coordinates (MNI space)** | |
| --- | --- | --- | --- |
| x | | **y z** | |
| Lingual_L | -14.62 | -67.56 | -4.63 |
| Occipital_Mid_L | -32.39 | -80.73 | 16.11 |
| Occipital_Sup_L | -16.54 | -84.26 | 28.17 |
| Occipital_Inf__L | -36.36 | -78.29 | -7.84 |
| Calcarine_L | -7.14 | -78.67 | 6.44 |
| Cuneus_L | -5.93 | -80.13 | 27.22 |
| Temporal_Mid_L | -55.52 | -33.80 | -2.20 |
| Postcentral_L | -42.46 | -22.36 | 48.92 |
| Precentral_L | -38.65 | -5.68 | 50.94 |
| Paracentral_Lobule_L | -7.63 | -25.36 | 70.07 |
| Occipital_Sup_R | 24.29 | -80.85 | 30.59 |
| Occipital_Mid_R | 37.39 | -79.70 | 19.42 |
| Occipital_Inf_R | 38.16 | -81.99 | -7.61 |
| Cuneus_R | 13.51 | -79.36 | 28.23 |
| Lingual_R | 16.29 | -66.93 | -3.87 |
| Paracentral_Lobule_R | 7.48 | -31.59 | 68.09 |
| Amygdala_R | 27.32 | 0.64 | -17.50 |
| ParaHippocampal_R | 25.38 | -15.15 | -20.48 |
| Hippocampus_R | 29.23 | -19.78 | -10.33 |
| Fusiform_R | 33.97 | -39.10 | -20.18 |
| Precuneus_R | 9.98 | -56.05 | 43.77 |
| Frontal_Sup_R | 21.90 | 31.12 | 43.82 |
| Frontal_Mid_R | 37.59 | 33.06 | 34.04 |
| Frontal_Inf_Orb_R | 41.22 | 32.23 | -11.91 |
| Frontal_Sup_Medial_R | 9.10 | 50.84 | 30.22 |
| Rectus_R | 8.35 | 35.64 | -18.04 |
| Cingulum_Ant_R | 8.46 | 37.01 | 15.84 |
| Cingulum_Post_R | 7.44 | -41.81 | 21.87 |
| Temporal_Inf_R | 53.69 | -31.07 | -22.32 |
| Temporal_Mid_R | 57.47 | -37.23 | -1.47 |
| Frontal_Sup_L | -18.45 | 34.81 | 42.20 |
| Frontal_Mid_Orb_L | -5.17 | 54.06 | -7.40 |
| Frontal_Inf_Orb_L | -35.98 | 30.71 | -12.11 |
| Cingulum_Ant_L | -4.04 | 35.40 | 13.95 |
| Rectus_L | -5.08 | 37.07 | -18.14 |
| Frontal_Mid_Orb_R | 33.18 | 52.59 | -10.73 |
